# Myocardial Expression Analysis of Osteopontin and Its Splice Variants in Patients Affected by End-Stage Idiopathic or Ischemic Dilated Cardiomyopathy

**DOI:** 10.1371/journal.pone.0160110

**Published:** 2016-08-01

**Authors:** Manuela Cabiati, Benedetta Svezia, Marco Matteucci, Luca Botta, Angela Pucci, Mauro Rinaldi, Chiara Caselli, Vincenzo Lionetti, Silvia Del Ry

**Affiliations:** 1 CNR Institute of Clinical Physiology, Pisa, Italy; 2 Laboratory of Translational Critical Care Medicine, Institute of Life Sciences, Scuola Superiore Sant’Anna, Pisa, Italy; 3 Department of cardiac Surgery, Niguarda Ca’ Granda Hospital, Milan, Italy; 4 Department of Pathology, University Hospital Pisa, Pisa, Italy; 5 Cardiac Surgery Department, Cardiothoracic Department, A.O.U. Città della Salute e della Scienza di Torino, Presidio Molinette, and University of Torino, Turin, Italy; University-Hospital of Parma, ITALY

## Abstract

Osteopontin (OPN) is a phosphoglycoprotein of cardiac extracellular matrix and it is still poorly defined whether its expression changes in failing heart of different origin. The full-length OPN-a and its isoforms (OPN-b, OPN-c) transcriptomic profile were evaluated in myocardium of patients with dilated or ischemic cardiomyopathy (DCM n = 8; LVEF% = 17.5±3; ICM n = 8; LVEF% = 19.5±5.2) and in auricle of valvular patients (VLP n = 5; LVEF%≥50), by Real-time PCR analysis. OPN-a and thrombin mRNA levels resulted significantly higher in DCM compared to ICM patients (DCM:31.3±7.4, ICM:2.7±1.1, p = 0.0002; DCM:19.1±4.9, ICM:5.4±2.2, p = 0.007, respectively). Although both genes’ mRNA levels increased in patients with LVEF<50% (DCM+ICM) with respect to VLP with LVEF>50%, a significant increase in OPN (p = 0.0004) and thrombin (p = 0.001) expression was observed only in DCM. In addition, a correlation between *OPN-a* and *thrombin* was found in patients with LVEF<50% (r = 0.6; p = 0.003). The mRNA pattern was confirmed by *OPN-a* cardiac protein concentration (VLP:1.127±0.26; DCM:1.29±0.22; ICM:1.00±0.077 ng/ml). The OPN splice variants expression were detectable only in ICM (**OPN-b**: 0.357±0.273; **OPN-c**: 0.091±0.033) and not in DCM patients. A significant correlation was observed between collagen type I, evaluated by immunohistochemistry analysis, and both OPN-a mRNA expression (r = 0.87, p = 0.002) and OPN protein concentrations (r = 0.77, p = 0.016). Concluding, OPN-a and thrombin mRNA resulted dependent on the origin of heart failure while OPN-b and OPN-c highlighted a different expression for DCM and ICM patients, suggesting their correlation with different clinical-pathophysiological setting.

## Introduction

Adverse structural remodelling leads to heart failure (HF) that is characterized by ongoing structural rearrangement in the architecture of the ventricular myocardium resulting in clinical and pathophysiological overt HF [1]. The two most frequent causes of end-stage HF in the developed countries are dilated (DCM) and ischemic (ICM) cardiomyopathy [2].

On the structural level, there are specific changes of ventricular geometry, intra and extracellular matrix (ECM) composition as well as cell and capillary loss and increased myocyte size as well [3]. All abovementioned phenotypic features are referred to as cardiac remodelling.

A key regulator of remodelling process in the heart is the ECM that consists of structural and non-structural proteins interacting via specific cell surface receptors or soluble growth factors and cytokines [4].

Osteopontin (OPN) is a typical phosphoglycoprotein of cardiac ECM often overexpressed in the human blood and remodelled myocardium during the development of HF [5]. Pre-clinical studies demonstrated that healthy murine myocardium expresses low levels of OPN in response to increased afterload [6] and in human, increased plasma levels of OPN are associated with activation of the renin-aldosterone system and with myocardial and coronary microvascular damage in dilated cardiomyopathy [7] but it is still poorly defined whether its expression changes in failing heart of different origin.

However, the myocardial role of OPN isoforms is still not well defined. Even though *OPN* is synthesized in various tissue and cell types, it is thought to be an inhibitor of vascular calcification expressed by calcifying vascular cells; it is also detectable in activated resident pericytes during angiogenetic processes, and has been found in fibro calcific plaques [8]. The myocardial OPN expression is increased during both acute and chronic disease [9]. OPN expression is increased in macrophages recruited in infarcted heart and cardiomyocytes are the major source of OPN in humans and rodents hypertrophied hearts [10].

In particular, the rise of OPN expression coincides also with the transition from myocardial hypertrophy to HF, and its level of expression is correlated with the severity of the HF [11–15]. Findings from several experimental studies have highlighted a link between the progression of cardiomyopathies (i.e.: DCM) towards HF and OPN expression [16–18]. However, the regulatory mechanisms underlie the OPN expression in the remodeled myocardium are still unknown. It is recognized that the tissue factor, the major trigger of blood coagulation, is involved in the onset of several cardiovascular diseases and that one of the ultimate events of the tissue factor initiated coagulation cascade is thrombin that is able to cleave OPN [19] suggesting the implication of these proteins in the pathophysiology of cardiac remodeling.

However, it is still poorly defined whether OPN and thrombin expression changes in failing heart of different origin. Moreover, OPN precursor mRNA undergoes alternative splicing leading to full-length OPN-a (i.e., consist of all exons), OPN-b (lacks exon 5) and OPN-c (lacks exon 4) as well [20]. The OPN splice variants are differentially expressed and may have functional heterogeneity in tumor specific manner [21] and recently are beginning to be studied in other diseases as calcific aortic valve disease [22], carotid atherosclerotic plaques [23] and systemic inflammatory conditions [24]. We have hypothesized that OPN isoforms and thrombin mRNA profile underlies the occurrence of pro-remodeling or pro-repair phenotype in end-stage failing heart of different origin. In fact, OPN is a component of perivascular and hematopoietic niche that suppresses the proliferation and function of hemopoietic stem and progenitor cells [25]. So far, no data are reported about myocardial OPN splice variants in different types of failing heart. The aim of our study was to measure the myocardial levels of OPN-a, thrombin, as well as of the two isoforms OPN-b and OPN-c in the left ventricle of patients affected by end-stage idiopathic or ischemic dilated cardiomyopathy.

## Materials and Methods

### Samples collection and RNA extraction

The investigation conforms to the principles outlined in the Declaration of Helsinki (Br Med J 1964; ii:177). The study was approved by the local *Ethical Committee of the San Giovanni Battista Hospital*, Turin Italy and all patients provided signed informed consent.

In the study were enrolled a total of twenty-one subjects. Sixteen HF patients were affected by end-stage idiopathic DCM (n = 8) or ICM (n = 8) and eligible for heart transplantation according to recent guidelines [26]. Patients with arterial hypertension, recent myocardial infarction (≤6 months before surgery), myocarditis or diabetes were excluded from the study. All patients received conventional therapy for end-stage HF and were not chronically treated with high-dose catecholamine infusions. The patients received similar dose of diuretics, beta-blockers and ACE-inhibitors. The patients were not affected by renal failure.

Idiopathic dilated cardiomyopathy was diagnosed on the basis of echocardiographically documented end-diastolic diameter >56 mm, left ventricular ejection fraction (LVEF %) <50% and normal coronary angiography. The selected ICM patients were affected by three-vessel coronary disease. We selected patients with known cardiomegaly of more than 6 months’ duration. All patients had waited a similar time on the waiting list for heart transplantation. Table 1 reports the baseline characteristics of HF patients.

**Table 1:** Biochemical characteristics of DCM and ICM patients.

	DCM PATIENTS (n = 8)	ICM PATIENTS (n = 8)
AGE (yrs)	42 ± 6	55 ± 2
GENDER, ♂, n (%)	8 *(100)*	8 *(100)*
LV EJECTION FRACTION, %	17.5 ± 3	19.5 ± 5.2
MEAN ARTERIAL PRESSURE, mmHg	76.7 ± 5	74.3 ± 4.7
SYSTOLIC ARTERIAL PRESSURE, mmHg	98± 5.54	96.5 ± 3
DIASTOLIC ARTERIAL PRESSURE, mmHg	66 ± 5.98	63.2± 2.5
LV END-DIASTOLIC VOLUME, ml	305.5 ± 110	270± 97
LV END-SYSTOLIC VOLUME, ml	255 ± 90	220 ± 88
SYSTEMIC VASCULAR RESISTANCE, mm Hg/min/l	18.5 ± 4.8	22 ± 10

To understand if OPN and thrombin expression depend on the origin of HF rather than the magnitude of global cardiac function, 5 patients who underwent surgical repair of heart valve disease and without concomitant coronary artery diseases (VLP, male, no pacemaker, no left bundle branch block, no pharmacological treatment), aged 50±3 years, with LVEF % >50% and LV end-diastolic diameter < 70mm were used.

Exclusion criteria were acute myocardial infarction or unstable angina within 6 months before the examination, significant primitive pulmonary disease and renal failure (defined as a serum creatinine value above 1.5 mg/dl).

In HF patients myocardial samples were harvested from the inter ventricular septum (IVS), which represents the LV region early remodelled during HF progression [27] in the beating dyssynchronous failing heart; in VLP samples were collected from auricle. Failing hearts were sampled at the time of heart transplantation; the VLP samples were collected during the valvular surgery. Both samples were immediately placed in ice-cold RNA*later* and stored at –80°C or snap frozen.

Total *RNA* was extracted by acid guanidinium thiocyanate-phenol-chloroform method from cardiac tissue samples with Rneasy Midi kit (Qiagen S.p.A, Milano, Italy) as previously describe [28–30]. The extraction methodology used was specific for fibrous tissues, as cardiac samples, and during extraction phases cellular debris were removed. RNA concentration was determined spectrophotometrically (Biophotometer reading, Eppendorf, Italy) at 260 nm. The ratio of readings at 260 nm and 280 nm (A_260_/A_280_) provided an estimate of the purity of *RNA* and only samples that showed OD 260/280 ratios of 1.9–2.1 were used. To check the total RNA integrity, all samples were also subjected to denaturating gel electrophoresis in order to visualize and evaluate the optical density of 18 S and 28 S rRNA bands. The RNA samples were stored at -80°C for use in gene expression studies.

### Reverse Transcription and Real-Time PCR

Following DNAse treatment, first strand cDNA was synthesized with iScript cDNA Synthesis kit (Bio-rad, Hercules, CA, USA) using about 1 μg of total RNA as template. Reverse transcriptase reaction sequence consisted of incubation at 25°C for 5 min, followed by three different cycles at 42°C for 30 min and 45°-48°C for 10 min, in order to better separate the strands. The reverse transcriptase enzyme was inactivated by heating to 85°C for 5 min. The cDNA samples obtained were placed on ice and stored at 4°C until further use. Real-Time PCR reactions were performed in duplicate in the Bio-Rad C1000 TM thermal cycler (CFX-96 Real-Time PCR detection systems, Bio-Rad) as previously described [30]. For monitoring cDNA amplification a third-generation fluorophore, EvaGreen, was used (SsoFAST EvaGreen Supermix, Bio-Rad). PCR was performed in a volume of 20 μl per reaction, including 0.2 μM of each primer (Sigma-Aldrich, St. Louis, MO, USA) samples, reagent and sterile H_2_O. Amplification protocol started with 98°C for 30 s followed by 40 cycles at 95°C for 5 s and 60°C for 30 s. Primer pairs of both interested markers and reference genes were designed with Primer Express Version 2.0 (Applied Biosystems) and details are given in Table 2. Two inter-run calibrators where used to ensure the comparability of different PCR runs. All reactions were performed in duplicate.

**Table 2:** Primer sequence details of the analyzed gene.

*Genes*	*Primer sequence*	*GenBan*, *accession n*.	*Length (pb)*	*Temp (°C)*	*Efficiency (%)*	*R*^*2*^
eEF1a	**F:** CTTTGGGTCGCTTTGCTGTT	NM_001402	183	60	101.7	0.998
	**R:** CCGTTCTTCCACCACTGATT					
RPL13a	>**F:** CGCCCTACGACAAGAAAAAG	NM_012423	206	60	104	0.999
	**R:** CCGTAGCCTCATGAGCTGTT					
RPS4X	**F:** GATCCCCTCATCAAGGTGAA	NM_002046	243	60	104.2	0.999
	**R:** GCCCTTGCCAATAACAAAAA					
OPN-a	**F:** AATGATGAGAGCAATGAG	NM_001040058	114	60	103	0.999
	**R:** GTCTACAACCAGCATATC					
OPN-b	**F:** CTGAGGAAAAGCAGCTTTACAAC	NM_000582	111	60	105	0.996
	**R:** ACTTACTTGGAAGGGTCTGTG					
OPN-c	**F:** GAGGAAAAGCAGAATGCTGTGT	NM_001040060	88	60	95.5	0.995
	**R:** GGTCATGGCTTTCGTTGGA					
THROMBIN	**F:** GAAGTGGATACAGAAGGTCAT	NM_000506	84	60	101.2	0.994
	**R:** TCTTTCACGGGATTGGTT					

***eEF1a***: Eukaryotic translation elongation factor 1 alpha 1; ***RPL13a*:** Ribosomal protein L13a; ***RPS4X*:** 40S ribosomal protein S4, X isoform; ***OPN-a*:** osteopontin full-lenght; ***OPN-b*:** osteopontin transcript variant 2 (or secreted phosphoprotein 1 (SPP1), transcript variant 2); ***OPN-c*:** osteopontin transcript variant 3 (or secreted phosphoprotein 1 (SPP1), transcript variant 3); ***THROMBIN*:** coagulation factor II

### Protein extraction

Tri-reagent procedure (Molecular Research Center, Cincinnati, OH, USA) allowed obtaining RNA and proteins from a single sample using a monophasic mixture of phenol and guanidine thiocyanate and isopropyl alcohol to precipitate nucleic acids, as previously described [29,30]. Proteins isolated by organic phase were added to ethanol and centrifuged to eliminate the lipid component. Subsequently, the addition of acetone and centrifugation (12,000 x *g*, 5 min at 4°C) made up a protein pellet that was washed and centrifuged three times with a wash buffer (guanidine, glycerol 25%, ethanol 96%). After a last wash with a solution of glycerol 25% and ethanol the pellet was re-suspended with Tris (hydroxy–methyl–aminomethane) HCl (4 mM) buffer (pH 7.4) [NaCl-154 mM, phenyl–methyl–sulfonylfluoride–PMSF-0.1 mM, sodium dodecyl sulfate (SDS) 2%]. The final protein preparations were frozen at -20°C and the protein concentration was determined according to the method of Lowry using BSA as a standard.

### OPN assay

OPN-a was directly measured in cardiac protein extracts by a specific enzyme immunometric assays (Osteopontin, human ELISA, DRG Diagnostic, GmbH, Germany). Each sample was assayed in duplicate. A control sample was assayed in each run for quality control.

### Histological and immunohistochemistry analysis

Five μm-thick sections of IVS samples, DCM and ICM respectively, were used for histological analysis, as previously described by us [31]. Serial slices were stained by Masson’s trichrome to assess myocardial architecture and fibrosis. The evaluation were performed in left ventricular mesocardial and sub-endocardial layers of both DCM and ICM patients and in a fragment of auricle of VLP subjects. For each sample, two independent observers evaluated a minimum of three serial sections. Photomicrographs were taken using a DFC480 digital camera (Leica Microsystem, Cambridge, UK). Specific immunostainings for human collagen type I (polyclonal antibody Santa Cruz Biotechnology, Germany) was performed.

### Data analysis

In an effort to provide greater transparency of our results between research laboratories, this study was carried out to conform to the *Minimum Information for publication of Quantitative Real-Time PCR Experiments* (MIQE) [32].

Ten reference genes were tested and GeNorm software was used to establish the most stably expressed gene, as described by Vandesompele et al. [33]. The geometric mean of the three most stably expressed genes (RPL13a, eEF1a, RPS4X) was used for normalization of mRNA expression results.

The relative quantification was performed by ΔΔCt method using Bio-Rad’s CFX96 manager software (CFX-96 Real-Time PCR detection systems, Bio-Rad Laboratories Inc., Hercules, CA, USA). When expression values were not normally distributed, the logarithmic transformation of data was used for statistical analysis. Differences between more than two independent groups were analyzed by Fisher’s test after ANOVA. Differences between two independent groups were assessed by unpaired t-test.

The results are expressed as mean ± SEM and p-value was considered significant when < 0.05. The association between different variables were assessed by linear regression test after logarithmic transformation, when necessary. All data were analyzed by using Statview 5.0.1 software released for Windows Statistical (SAS Institute, Inc., Cary, NC, USA).

## Results

### OPN-a,-b, -c and thrombin expression

**Fig 1:**
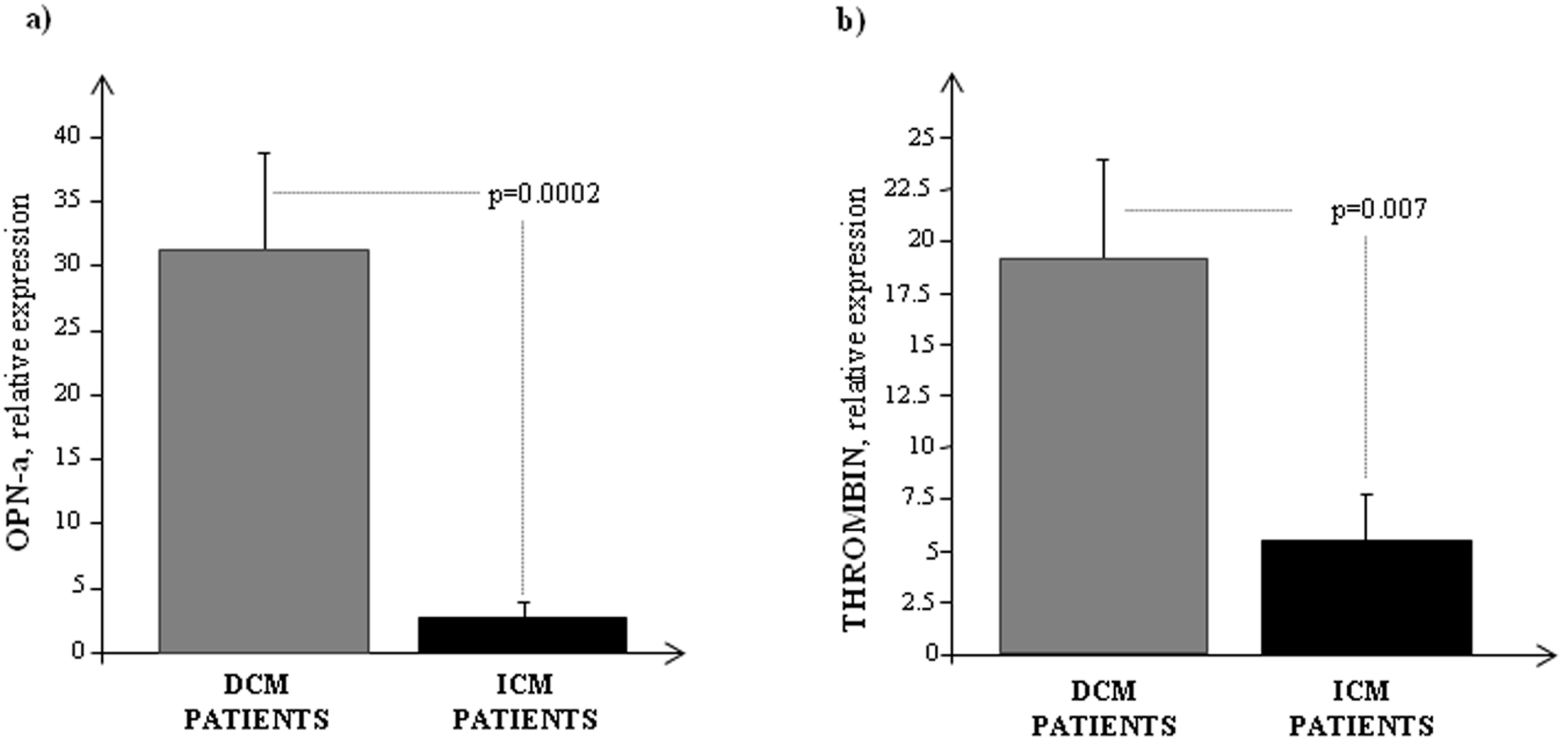
Transcriptional profile of OPN-a and thrombin in the heart of failing patients. a) OPN-a and b) thrombin mRNA expression measured by Real Time PCR in DCM and ICM patients. The three most stably expressed genes (*RPL13a*, *eEF1a*, *RPS4X*) was used for normalization of mRNA expression results.

As reported in Fig 1a, OPN-a mRNA levels were significantly higher in DCM compared to ICM patients. Similarly, the thrombin mRNA was highly expressed in end-stage DCM hearts (Fig 1b).

**Fig 2:**
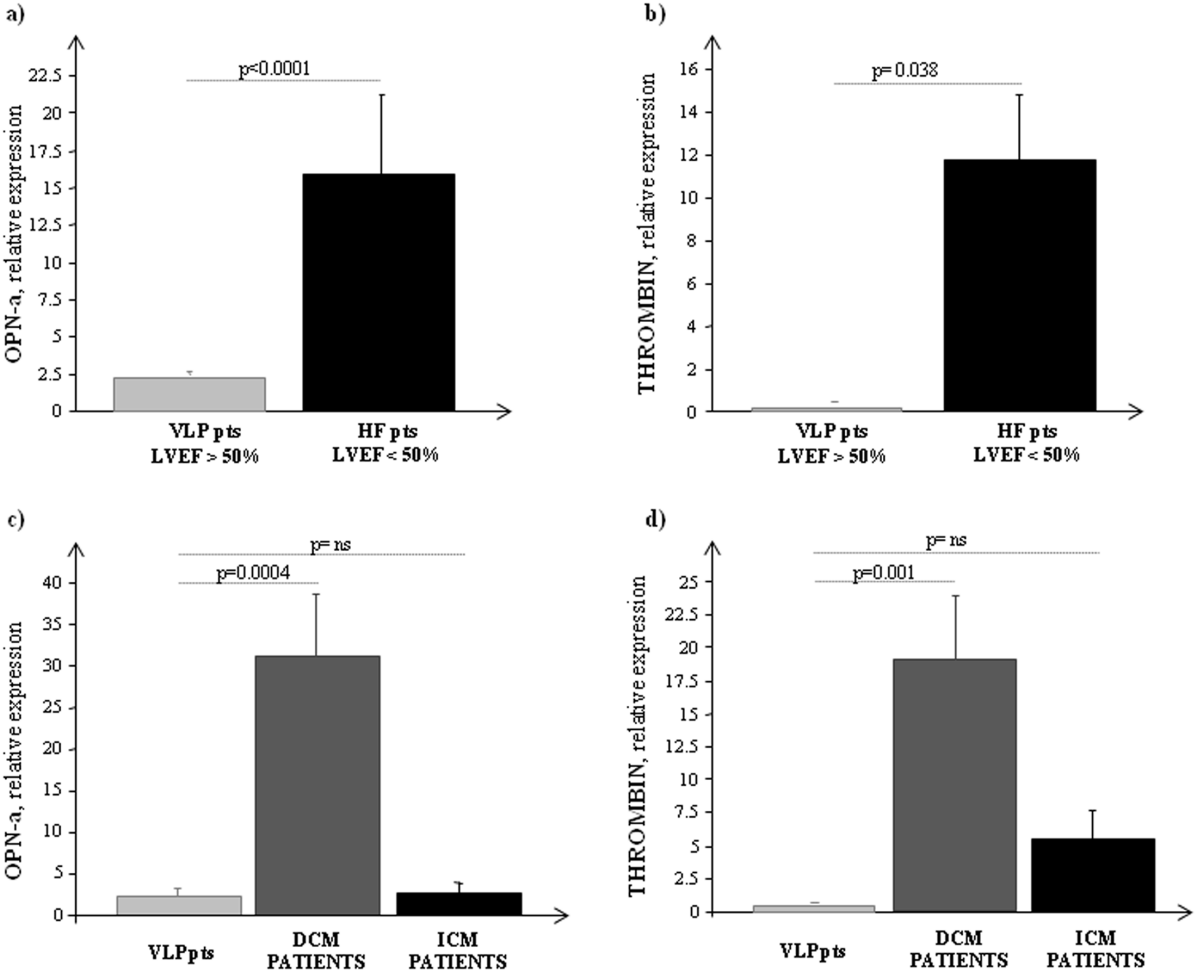
Transcriptional profile of OPN-a and thrombin in all group of patients studied. a) OPN-a and b) thrombin mRNA expression measured by Real Time PCR in VLP patients with LVEF>50% and HF patients with LVEF<50%; c) OPN-a and d) thrombin mRNA expression measured by Real Time PCR in VLP patients with LVEF>50% and HF patients splitting in DCM and ICM. The three most stably expressed genes (*RPL13a*, *eEF1a*, *RPS4X*) was used for normalization of mRNA expression results.

In order to understand if OPN and thrombin expression were dependent on the origin of HF rather than the magnitude of global cardiac function, their expression was analyzed in VLP with LVEF>50% and compared with the whole group of failing patients (DCM+ICM) with LVEF<50%. As reported in Fig 2 both biomarkers increased in HF patient with LVEF<50% with respect to those with normal global cardiac function (VLP). Interestingly when data belonging to HF patients were splitting in DCM and ICM, a significant increase in OPN and thrombin expression was observed only in DCM with respect to both VLP and ICM (Fig 2c and 2d).

A significantly positive correlation between OPN-a and thrombin was observed in HF patients (r = 0.6; p = 0.003) but not in those with LVEF>50% (VLP).

**Fig 3:**
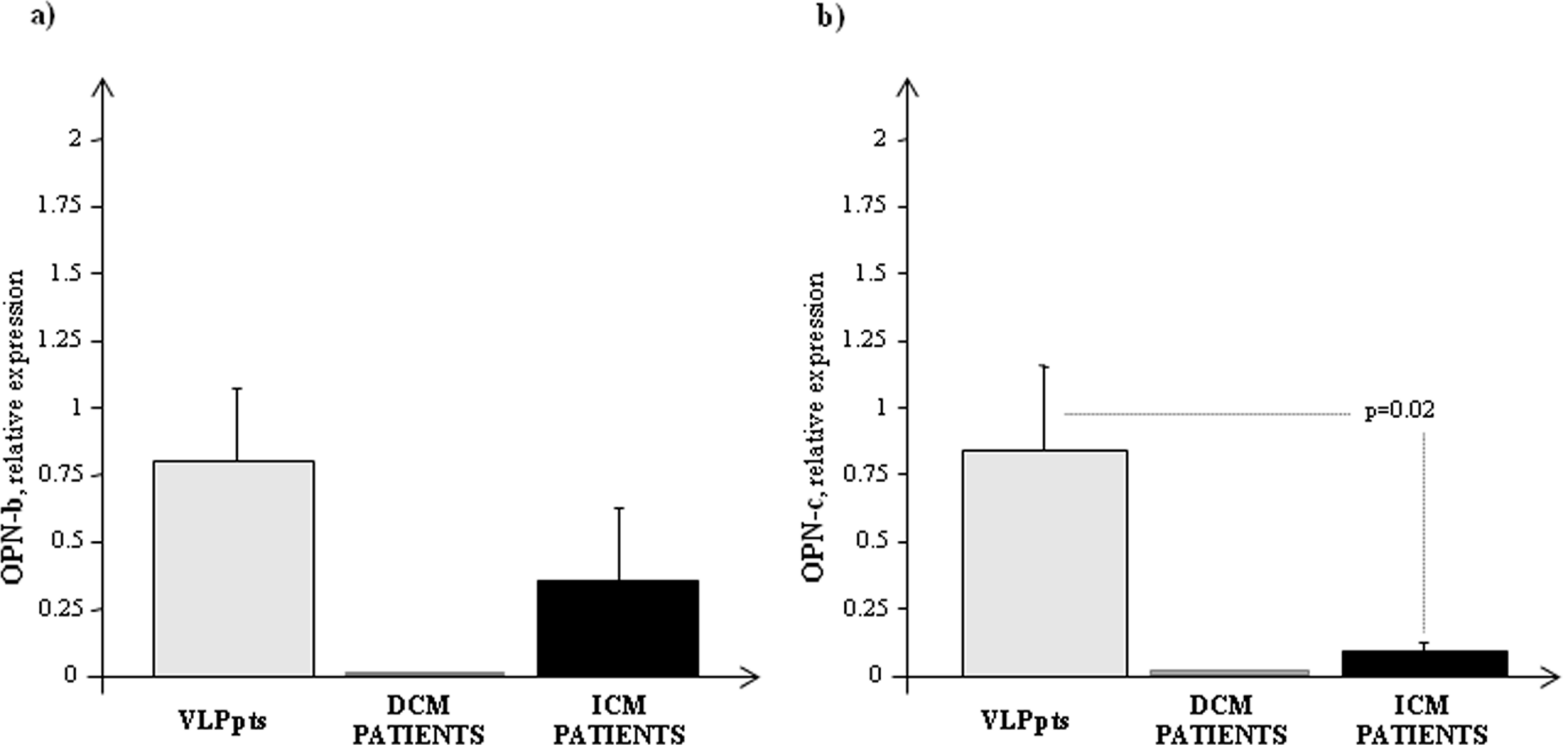
Transcriptional profile of OPN- b and -c in all group of patients studied. a) OPN-b and b) OPN-c mRNA expression measured by Real Time PCR in in VLP patients with LVEF>50% and HF patients splitting in DCM and ICM. The three most stably expressed genes (*RPL13a*, *eEF1a*, *RPS4X*) was used for normalization of mRNA expression results.

The OPN splice variants mRNA expression, OPN-b and OPN-c, were detectable only in ICM patients (**OPN-b**: 0.357±0.273; **OPN*-c***: 0.091±0.033), but not in DCM patients (Fig 3). As expected, the auricular myocardium, belonging to patients with valvular disease and normal atrial size, showed both isoforms mRNA expression (Fig 3).

### OPN-a immunometric determination

**Fig 4:**
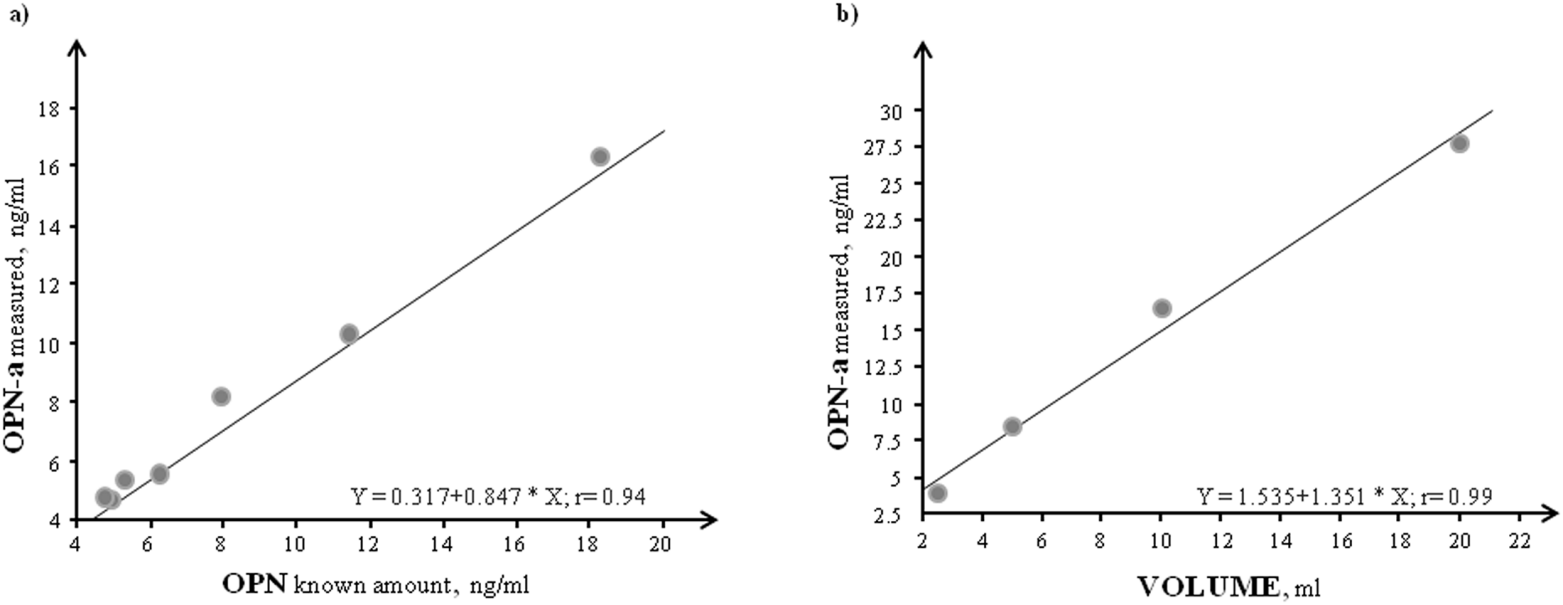
Methodological evaluation of OPN assay. a) recovery test, evaluated adding known amounts of *OPN* standard (0–32 ng/ml, 1:10 dilution) to a plasma pool; b) dilution test, carried out using serial dilution of plasma pool.

Table 3 reported the OPN assay analytical performance related to sensitivity, inter and intra-assay variability, obtained by repeated determinations of a plasma sample. The accuracy of the immunometric determination was evaluated by dilution and recovery tests as reported in Fig 4. OPN-a extract cardiac tissue levels were measured and the pattern observed at the mRNA level was confirmed by OPN-a cardiac protein concentration (VLP: 1.127±0.26; DCM: 1.29±0.22; ICM: 1.00±0.077 ng/ml).

**Table 3:** Analytical performance of OPN assay.

	INTRA-ASSAY VARIABILITY	INTER-ASSAY VARIABILITY	SENSITIVITY
**OPN**, ng/ml	2.58 ± 0.05 CV = 4.8%	2.64 ± 0.13 CV < 20%	0.6 ± 0.05

**CV** = Coefficient of Variation

**Fig 5:**
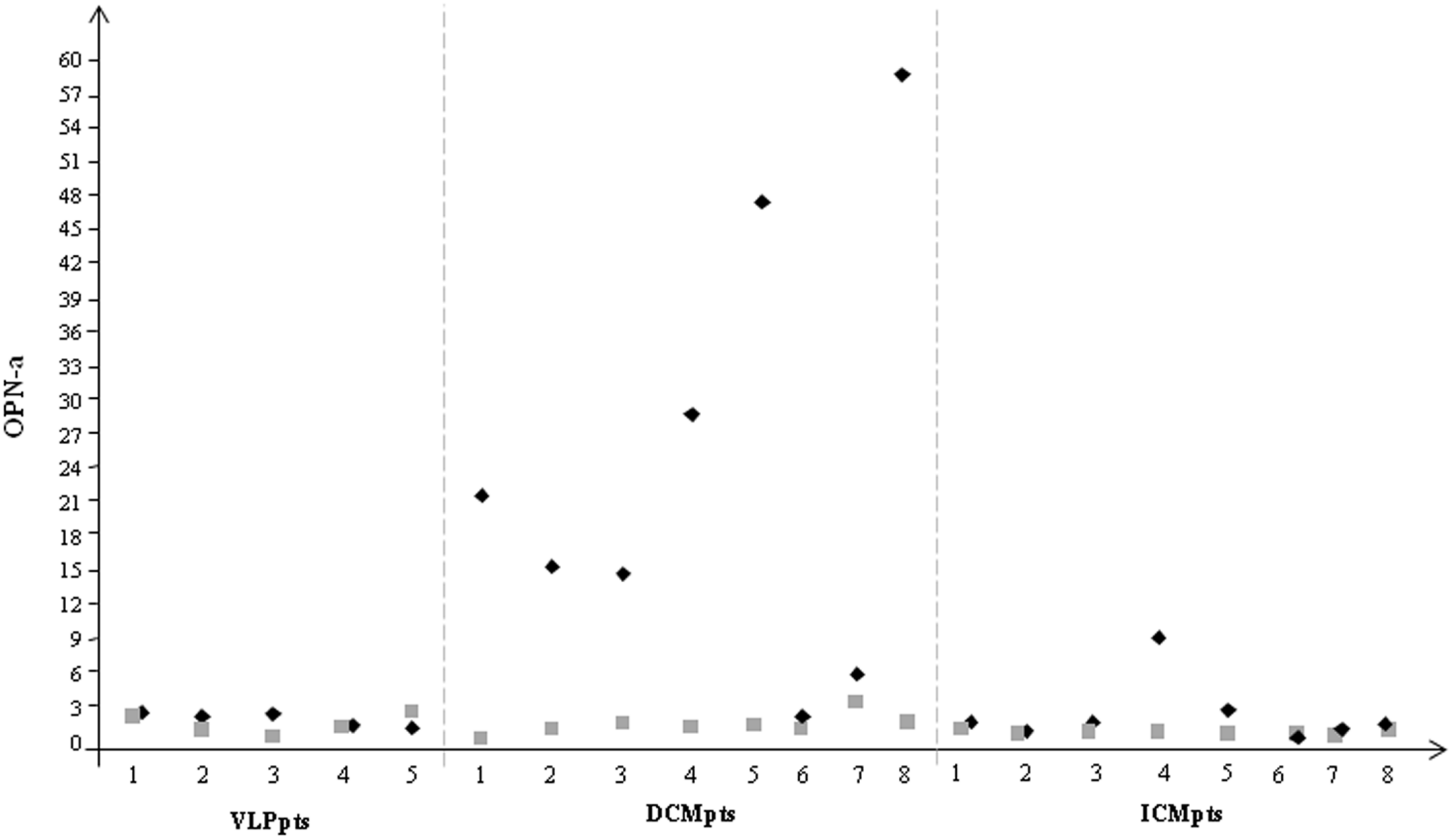
Comparison of OPN-a at protein and at mRNA level. Individual data plot of mRNA expression and protein concentration for VLP, DCM and ICM groups (Black rhombus:mRNA espression, grey square:protein concentration, ng/ml).

To underline the relationship between the mRNA expression and protein data in each singular patient, a plot of the individual results was showed for VLP, DCM and ICM groups in Fig 5. For each sample the mRNA expression resulted higher with respect to protein concentration mainly in DCM patients.

### Histological and immunohistochemistry analysis

**Fig 6:**
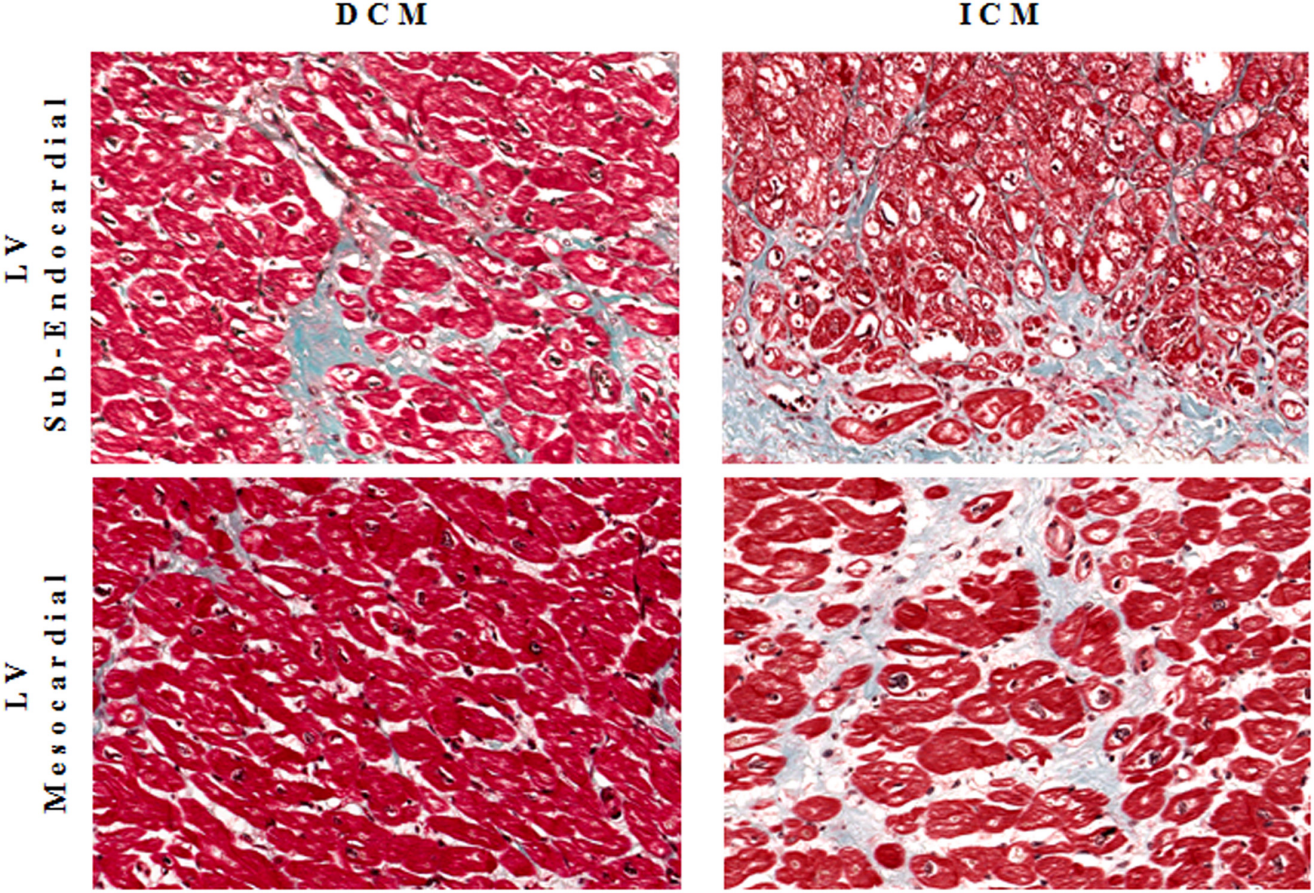
Histology. Histological representation (Masson’s trichrome) of left ventricular mesocardial and sub-endocardial layers in DCM (left) and ICM (right).

**Fig 7:**
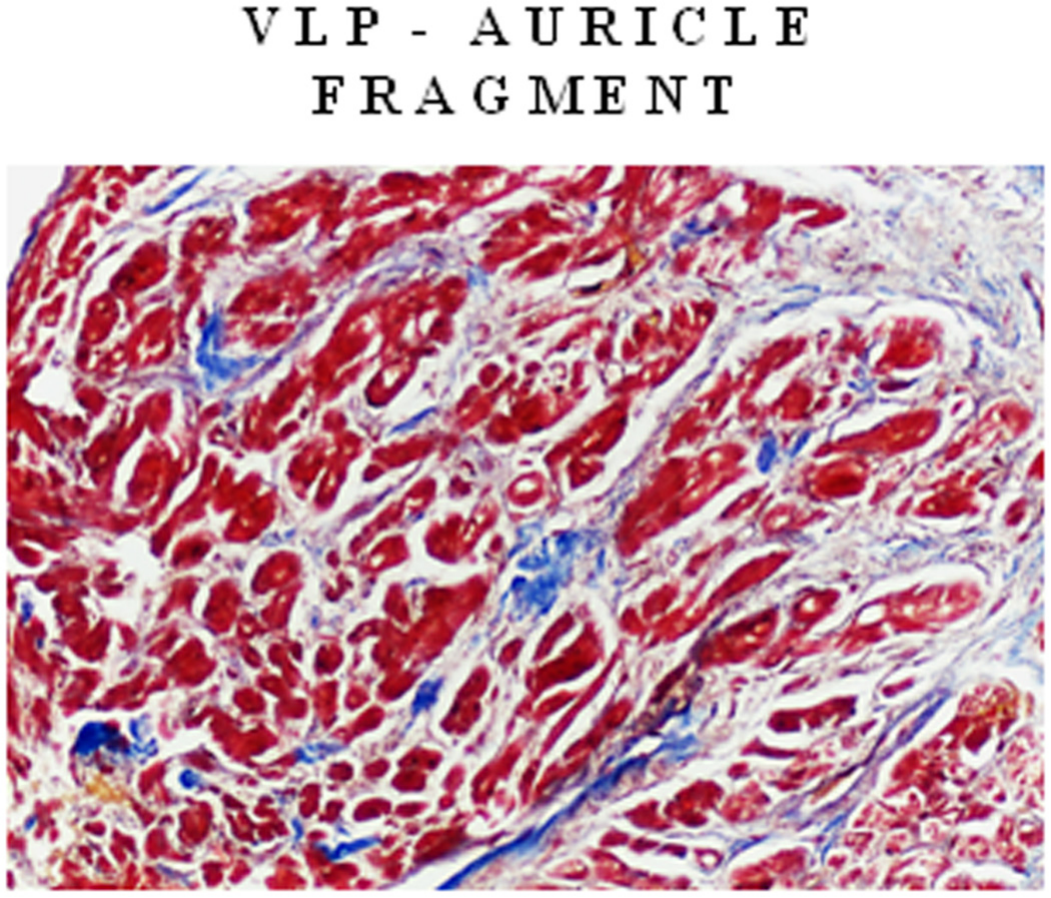
Hystology. Histological representation (Masson’s trichrome) of auricle in VLP.

As showed in Fig 6 a larger myocardial amount of type I collagen, fibronectin and fibroblasts was detected in ICM rather than DCM hearts where they were finely distributed in left ventricular mesocardial and sub-endocardial layers. A significant correlation was observed between collagen type I expression, previously measured by us in the same tissue samples [31], and both OPN-a mRNA expression (r = 0.87, p = 0.002) and OPN protein concentrations (r = 0.77, p = 0.016). As showed in Fig 7, fibrosis was undetectable in VLP myocardium.

## Discussion

Our study has demonstrated that changes of left ventricular OPN-a (full-length form) and thrombin mRNA expression were dependent on the origin of heart failure rather than on the type of medications, magnitude of contractile failure and microenvironmental features. At similar LVEF and ischemic microenvironment, the gene expression of OPN-a and thrombin was higher in DCM rather than ICM left ventricle.

It is conceivable that the greater loss of cardiomyocytes due to more widespread ischemic insult has reduced the capacity of the left ventricular myocardium to express OPN-a and thrombin mRNA in ICM hearts. Taking into account the known role played by OPN-a in ECM turnover activation, it is noticeable that the levels of myocardial OPN-a expression in failing ICM hearts were similar to those with normal systolic function and without myocardial fibrosis (VLP samples). Therefore, we suppose that OPN-a may be a novel biomarker of active ECM turnover, a remodeling signal in cardiomyocytes and also a predictor marker of cardiac failure of stem/progenitor cell function in failing hearts [25].

In our study, all data further suggest the hypothesis that cardiomyocytes-derived OPN-a is more expressed in the presence of still active ECM turnover, such as in end-stage DCM hearts where the segmental replacement fibrosis is lacking [31]. We have observed that levels of OPN-a were inversely related to myocardial fibrosis in the presence of similar decay of systolic function. We have detected larger myocardial deposits of type I collagen, fibronectin and fibroblasts in ICM rather than DCM hearts [31], in accord with previous studies [34–38]. Our data are also consistent with proteomic analysis performed by us on the same LV samples [31], which show the up-regulation of pro-apoptotic and pro-fibrotic factors in ICM failing hearts with overt fibrosis (ICM). In addition, we argue that the proteolytic cleavage of OPN-a in DCM samples may be independent on thrombin, which levels are similarly increased to those of OPN-a. Thus, other cleavage factors may be involved. Interestingly, Kostin et al [39] have demonstrated that human DCM failing hearts show 2-fold downregulation of cathepsin-D expression. Cathepsin D, an aspartyl lysosomal protease, is able to cleave OPN-a at the level of RGD domain [40], an arginine-glycine-aspartic acid domain that is also recognized by thrombin and plasmin [40], cell surface integrins expressed by fibroblasts [41] or non-RGD integrins, such as α4β1 and α9β1 [3, 42,43]. In our samples, the LV cathepsin-D expression was up-regulated in ICM rather than DCM failing hearts [31]. The above mentioned proteomic findings well support the detection of higher levels of OPN-a in the presence of higher thrombin levels in our DCM failing hearts. Therefore, thrombin may be considered only an hallmark of myocardial pro-inflammatory response, in accord with previous study [44].

Finally, we have also shown a different trancriptomic profile of myocardial OPN splice variants, OPN-b and -c, in DCM and ICM hearts with similar global cardiac function. Even if myocardial OPN-b and–c were undetectable in DCM patients, the gene expression of OPN variants in ICM failing myocardium was lower than VLP tissue, used as normal tissue. Our data are in accord with other study showing that cardiovascular OPN-b and–c mRNA may be inversely expressed compared to OPN-a [45].

Even if we don’t have data on protein detection of OPN-b and -c isoforms, we have first proof of modulation of myocardial OPN mRNA alternative splicing in failing myocardium, which is one of the main post-transcriptional modifications. In fact, the alternative splicing is the major mechanism of generating protein diversity from a limited amount of DNA and it is difficult to assess by Western blotting. It is an important mechanism to increase structural and functional diversity of proteins and the splicing variants deriving from this gene mechanism could provide diagnostic and or therapeutic targets for several pathologies.

### Conclusions

We have examined, for the first time, the expression profile of thrombin and splice variants OPN-a, -b and–c mRNA in the failing human LV myocardium of different origin. We have observed that OPN isoforms and thrombin mRNA levels are related to the aetiology rather than the magnitude of LVEF reduction and of fibrosis in end-stage failing heart. In addition, the OPN-a levels are independent on thrombin mRNA myocardial levels. Finally, levels of OPN-b and–c mRNA are reduced in the presence of high OPN-a levels. Further studies conducted in a large population cohort will be necessary to assess the reliability of OPN splice variants levels as hallmarks of ECM turnover or repair activated by cardiomyocytes in different clinical setting.

### Limitations

Even if previous study has demonstrate that there is a high transcriptional similarity between left ventricular and right atrial myocardial samples [46], one of the main limitations of our study is the lacking of IVS samples of normal subjects to be used as control tissue. Undoubtedly, direct measurement of the OPN-a and thrombin mRNA in normal tissue collected from human IVS septum would be ideal, but the sampling of LV tissue from normal human hearts raises major ethical concerns. In addition, the normal donor hearts unsuitable for transplantation are not proper control tissue as frequently exposed to high dose of catecholamines before the explantation.

The lack of additional tissue from IVS samples limited the analysis of OPN-b and -c protein levels, while the detection of changes in myocardial expression of these genes remains original.

Finally, the enrolment of a larger group of patients will be helpful to further strengthen our conclusions even if the differences between our data are already statistically relevant. Further investigations will better define the relationship between myocardial and plasmatic levels of OPN splice variants in HF patients of different origin.
